# The development of a legal framework for blood donation and blood safety in China over 24 years

**DOI:** 10.1186/s12913-020-05944-6

**Published:** 2020-11-30

**Authors:** Dajun Gao, Heng Li, Kang Wang

**Affiliations:** 1 Shanghai Jiao Tong University School of Medicine, Shanghai, China; 2 School of Public Health, Shanghai Jiao Tong University School of Medicine, Shanghai, China; 3 Law School, Shanghai University of Political Science and Law, Shanghai, China

**Keywords:** Voluntary non-remunerated blood donation, China, Transfusion, Legislation, NAT

## Abstract

**Background:**

This study analyzes the regulation of and developments in blood donation in China from 1996 to 2019, and demonstrates the government’s efforts to improve blood safety.

**Results:**

Since the implementation of the Blood Donation Law in 1998, the number of blood donors in China increased by 275% from 1998 to 2018 (from 4 million to 15 million). The principle of no-fault liability was proposed and has been applied since 2010 to the tort liability related to blood transfusion malpractice. In 2015, mutual blood donation accounted for 4.2% of the national collection. However, in some provinces of China, the percentage of mutual blood donation increased from 9.3 to 35.6% in 2016. The National Health Commission canceled mutual blood donation in March of 2018. Since 2015, nucleic acid amplification testing has become a routine test item for screening blood.

**Conclusions:**

The Chinese government institutionalized the voluntary non-remunerated donation principle, enacted regulations for the management of blood transfusion, and adopted advanced blood testing technology to sustain blood supply and ensure blood safety. Despite increased blood donation, blood shortages persist. The quality and safety of blood collection can be further improved through the cancellation of mutual blood donation and incentive measures for voluntary non-remunerated donation of blood, which needs facilitation by governmental legislation.

## Background

From the 1980s to early 1990s, the shortage of blood and the introduction of new plasma collection technology in China created a big opportunity for paid donation, targeting the poor in rural areas. Blood with HIV/AIDS, hepatitis C(HCV), and malaria, was common then, as were frequent cross infections [1, 2]. Disorderly pricing, weak supervision, and negligence of tests directly resulted in hepatitis B (HBV) and HIV epidemics, which were transmitted through blood collection and transfusion in some regions [3, 4]. In the mid-1990s, an outbreak of HIV infection among paid plasma donors in Anhui and Henan Provinces in China was confirmed [5–7]. Blood safety required more attention and improvement in China, and the Chinese government gradually adopted a series of legislations and policies to improve blood safety.

To solve the problem of blood safety, China implemented the Law of the People’s Republic of China on Blood donation (Blood Donation Law) in 1998, and institutionalized the principle of voluntary non-remunerated blood donation (VNRBD) by learning from international experience. VNRBD is an important measure for ensuring blood quality and safety. Blood safety has undergone significant changes in China since 1998. Currently, most scholars have reached a consensus regarding VNRBD and its foundation for a safe and sustainable blood supply [8]. In a review published by the World Health Organization (WHO), experts declared that without a system based on VNRBD, no country can provide sufficient blood for all patients who require transfusion [8]. China had a total of 459 blood centers in 2010, including 355 specialized blood stations and 104 blood stations in hospitals [9]. The number of blood donations and the volume of blood collection in China have been on the rise for 20 consecutive years [10]. In 1998, the number of unpaid blood donors nationwide was around 4 million, reaching nearly 15 million in 2018. In 1998, the volume of national blood collection was nearly 5 million units, reaching 25 million units by 2018 [11]. According to the WHO’s *2016 Global Status Report on Blood Safety and Availability*, apart from increased VNRBD, the number of blood donations, and volume of blood collection, blood safety has been improved by the blood nucleic acid test (NAT) technology adopted by China; and the clinical, rational use of blood has likewise significantly improved in China [12]. Despite more than 20 years of continuous improvement, there are still many shortcomings in blood safety in China, such as the increasing need for blood and blood products, the risk of transfusion-transmitted infections that lead to chronic blood shortages, unsafe blood products, and unsound clinical transfusion practices [13–18].

The recent status of blood donation and transfusion in China as per data from 1998 to 2019 is presented in this study. Furthermore, improvements and developments in blood donation mode, legal framework, policy, screening technology, and government management are demonstrated. The legal framework and policies on blood donation and transfusion have shown to be effective intervention mechanisms in ensuring blood quality and safety for more than 20 years. Development of the blood donation system in China is not only a reference model for other developing countries, but also an indicator of blood donation system problems and trends to help improve blood safety and availability in China.

## Methods

The laws and regulations related to blood donation and transfusion that were implemented or revised from 1998 to 2019 in China were consolidated to expound on the current legal framework of blood transfusion and the roles of the government, blood stations, and hospitals in the management of clinical blood use. Documents on the laws, regulations, and notices were collected from official Chinese government websites, and the data on blood donation were obtained from official reports of the National Health Commission (NHC) from 1998 to 2019, the WHO’s Global Database on Blood Safety from 1998 to 2011, and the 2016 Global Status Report on Blood Safety and Availability. We used “blood transfusion,” “medical damage liability,” “HBV,” “HIV,” “HCV,” and “syphilis” as keywords to search litigation on the China Judgements Online Chinese court documents website. A total of 484 related cases were downloaded, and 301 of them qualified after screening.

## Results

### The change of blood donation modes from paid and mobilized unpaid to voluntary unpaid blood donation

**Fig. 1:**
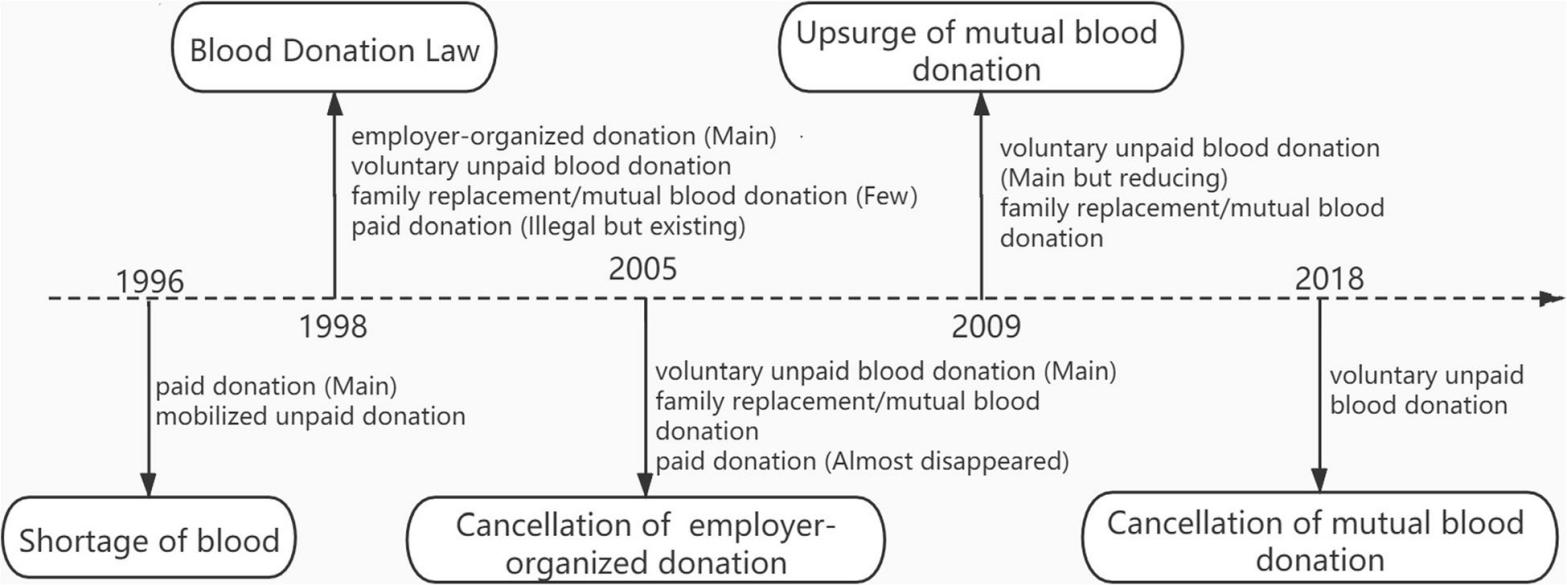
Important policies in the development of China’s blood donation modes

In 1998, the Blood Donation Law became a symbol of the VNRBD system in China. VNRBD, family replacement/mutual blood donation (FRMBD), and employer-organized blood donation were the three types of blood donation programs permitted by the Blood Donation Law (see Fig. 1).

Employer-organized blood donation is an interim policy from paid blood donation to voluntary unpaid blood donation. In 2005, employer-organized blood donation, workers’ blood donation as pre-arranged by the employer, and local blood stations, a semi-obligatory and mobilized unpaid mode, were banned by the Chinese government [19, 20]. The reason for the ban was that those who donated blood, as required by their employer, were exposed to undue pressure and had higher rates of infectious disease markers compared to volunteer donors [21–24]. In fact, the cancellation of employer-organized blood donation meant that China implemented a voluntary unpaid donation system nationwide, thereby eliminating paid donation by 2005. Of course, group voluntary blood donation, as a mode of VNRBD, is allowed in China, accounting for a large percentage of blood donation. Group blood donation has the advantage of being arranged in advance, making up for seasonal shortages in street blood collection and emergency mobilization [25].

There are three identified types of blood donors in the WHO Blood Safety and Availability Report: voluntary unpaid, family or replacement, and paid [12]. FRMBD is concerned with patients’ family members, relatives, and friends – the unit to which he or she belongs in the community – under blood donation for mutual aid, thereby ensuring the supply of blood for citizens’ clinical first-aid treatment [26]. FRMBD has been widely used for more than 20 years in China. Since 2009, the government of China has been giving increased attention to the proportion of FRMBD in blood collection. According to the NHC, the policy of FRMBD was canceled in 2018 across most regions, to improve blood quality and safety [27].

### Roles of the government, blood centers, and medical institutions in the management of blood collection and transfusion

Blood must be used for official clinical activities. Any form of blood trade has been banned by the Chinese government to assure the quality and safety of blood collection, which were listed in accordance with blood-related laws and regulations (see Table 1). (From 1996 to 2013, China’s National Health Administration agency was The Ministry of Health, and it was revoked and replaced by The National Health and Family Planning Commission from 2013 to 2018. Since March 2018, it has been replaced by the NHC). Even the importation and exportation of blood was forbidden in 2017 [28]. To encourage donation, the government provides voluntary blood donors with a holiday, a nutrition allowance, and priority access to blood transfusion during emergencies [29].

**Table 1:** The Outline of Laws and regulations related to Blood Donation and Safety in China

Type	Act	Legal sources	Implementation Years	Legislature	Main points
Specific Legislations	Regulations on Blood Collection and Supply Institution and Blood Administration	Regulation	1993 (1998 Expired)	The Ministry of Health^a^	Permission of The Blood Centre Blood Donor Registration Promotion of VNBD
	Detailed Rules for the National Verification of External Immunodiagnostic Reagent for Blood Use	Regulation	1994	The Ministry of Health	Test of HIV, HBV, HCV, Syphilis
	Regulations on Administration of Blood Products	Regulation	1996(2016 Revision)	Instrumentalities of the State Council	Blood Product Administration
	Blood Donation Law	Law	1998	Standing Committee of the National People’s Congress	VNBD System; Blood Only for Clinical Use
	Measures for the Administration of Blood Centres (for Trial Implementation)	Regulation	1998 (2006 Expired)	The Ministry of Health	Blood Centre Administration
	regulations on Clinical Use of Blood in Medical Institutions (for Trial Implementation)	Regulation	1999 (2012 Expired)	The Ministry of Health	Clinical Blood Use
	Technical Standards for the Clinical Blood Transfusion	Regulation	2000	The Ministry of Health	Corss-Match Test Blood Transfusion Record
	Measures for the Administration of Blood Centres	Regulation	2006 (2009, 2016, 2017 Amendment)	The National Health and Family Planning Commission^a^	Classification and Management Of Blood Centre Blood Specimen Restoration 2017 Cancellation of Blood Imports and Exports
	Provisions on Clinical Use of Blood in Medical Institution	Regulation	2012	The National Health and Family Planning Commission	Clinical Blood Use Emergency Blood Use
	Technical Operating Procedures for Blood Centres (2019 Edition)	Regulation	2019 Edition (2005, 2012, 2015 Edition Expired)	The National Health Commission^a^	NAT in clinical use
Other Relative Legislations	Regulation on the Handling of Medical Accidents	Regulation	2002	Instrumentalities of the State Council	Fault Compensation Liability
	Tort Law	Law	2010	Standing Committee of the National People’s Congress	Principle of No-Fault Liability in Blood Transfusion Tort
	Pharmaceutical Administration Law	Law	1984 (2001, 2002, 2004, 2016, 2017, 2019 Revision) (2013 Amendment)	Standing Committee of the National People’s Congress	Blood Products Blood Products Cannot Be Commissioned Production and Sell Online
	Prevention and Treatment Of Infectious Diseases Law	Law	1989 (2004 Revision) (2013 Amendment)	Standing Committee of the National People’s Congress	Ensure the Quality Of Blood And Blood Products To Prevent Transfusion-Transmitted Diseases
Local Legislation	Regulations on Voluntary Blood Donation By Citizens Of Beijing	Regulation	1992 (1998 Expired)	Beijing’s Standing Committee of the National People’s Congress	Promotion of voluntary blood donors
	Regulations on Shenzhen Special Economic Zone on Citizen’s Gratis of Blood Donation and Blood Management	Regulation	1995 (2015 Expired)	Shenzhen’s Standing Committee of the National People’s Congress	Reimbursement of VNBD
	Regulations of Beijing Municipality on Mobilizing and Arranging for Citizens to Donate Blood	Regulation	1998 (2006 Expired)	Beijing’s Standing Committee of the National People’s Congress	VNBD system
	Rules of Guangzhou Municipality on Donation of Blood	Rules	2004 (2015 Amendment)	Guangzhou Municipal People’s Government	Mutual Blood Donation
	Regulations of Nanning Municipality on Blood Donation	Regulation	2004 (2012 Revision)	Nanning’s Standing Committee of the National People’s Congress	VNBD system
	Measures of Beijing Municipality for Administration of Blood Donation	Rules	2009	Beijing Municipal People’s Government	VNBD publicity and the service
	Regulations on Shenzhen Special Economic Zone Blood Donation	Regulation	2015 (2019 Amendment)	Shenzhen’s Standing Committee of the National People’s Congress	VNBD incentives
	Measures of Nanning for rewarding blood donation	Rules	2017	Standing Committee of Nanning Municipal People’s Government	VNBD incentives and rewarding

^a^From 1996 to 2013 the national health administration department of China is The Ministry of Health, and it was revoked and replaced by The National Health and Family Planning Commission from 2013 to 2018. Since March 2018, it was replaced by The National Health Commission

Blood centers serve as the main institution in the collection of blood. From 1993 to 1998, the establishment of blood centers has been consistently approved by the Red Cross Society in China [30]. After the *Measures for Blood Center Administration (for Trial Implementation)* were implemented in 1998, the blood center was defined as a nonprofit, public welfare organization, and its establishment was thereafter managed by the health administration department of the provincial government [31]. Blood centers provide the necessary health examination and blood collection service for voluntary unpaid blood donors, maintain blood supply for clinical use, and are responsible for storage and transportation [32]. Blood collected from donors is tested to avoid quality problems [33].

The medical institution is the only legal institution for the clinical use of blood. The Blood Donation Law stipulates that all blood and blood products must be tested before transfusion in medical institutions to ensure safety [34]. Other regulations and technical standards regarding blood centers and medical institutions are listed in Table 1. These guidelines and regulations have the effect of strengthening the management of blood collection and enhancing the level of blood safety.

### Significant increase in blood collection and supply

Before 1998, the recruitment of blood donor volunteers was a very challenging endeavor in China. Traditional Chinese medicine holds that the loss of even a small amount of blood was harmful to health; this was also why paid blood donation was common at the time [3].

**Fig. 2:**
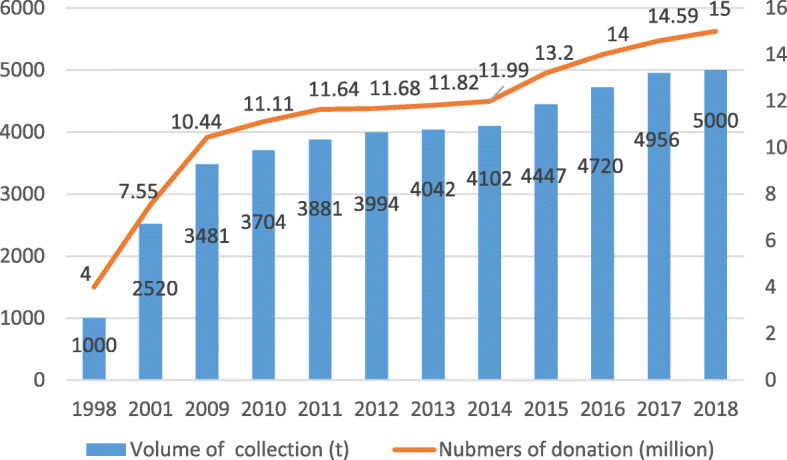
Numbers of Donors & Volume of Donation from 1998 to 2018

Since the 1998 implementation of the Blood Donation Law, and encouragement of unpaid blood donation through laws and policies [35], the number of unpaid blood donors and the amount of blood collected in China have been continuously increasing for 20 years (Fig. 2). (Data on 1998, 2010, 2011, 2014, 2015, 2016, 2017, 2018 from the NHC. Data on 2012 and 2013 from the Global Database on Blood Safety). The steady increase in the number of unpaid blood donors and the amount of blood collected has ensured the safe supply of blood from the source [11].

The proportion of unpaid blood donation, which was only 8% in 1998, increased to 95.5% in 2005. After 2009, all clinical blood came from unpaid donation [36]. In response to the call of The Melbourne Declaration on 100% VNRBD and Blood Components, the Chinese government engaged in efforts to popularize VNRBD donors and joined the list of countries which reported almost 100% blood collection in 2011 [37].

Through the establishment of a multi-level alarm mechanism when blood is in short supply, and the deployment of resources in different blood centers, seasonal, regional, and partial blood donation problems have been solved to a great extent. In 2015, 1.19 million units of blood were allocated across the country, reaching 1.54 million in 2017 [38]. In 2018, a total of 1.84 million units of blood were allocated across the country, of which 1.585 million units (86.1%) were allocated between the cities in the same province and 255 thousand units (13.9%) were allocated between provinces. The policy of raising an alarm regarding the need for blood and deploying it effectively ensured clinical blood supply in need-intensive areas and for major public health events [11].

### The rise and fall of FRMBD in VNRBD

Since 1998, according to Article 15 of the Blood Donation Law, patients’ family members, relatives, friends, and colleagues have been allowed to donate blood for mutual aid in emergency situations. FRMBD is a double-edged sword for blood donation. On the one hand, it can solve the shortage of blood. On the other hand, the risk of blood trade exists in mutual blood donation. The WHO has stressed that when mutual blood donation accounts for more than 5% of unpaid blood donation, there is a risk of illegal blood trade.

**Fig. 3:**
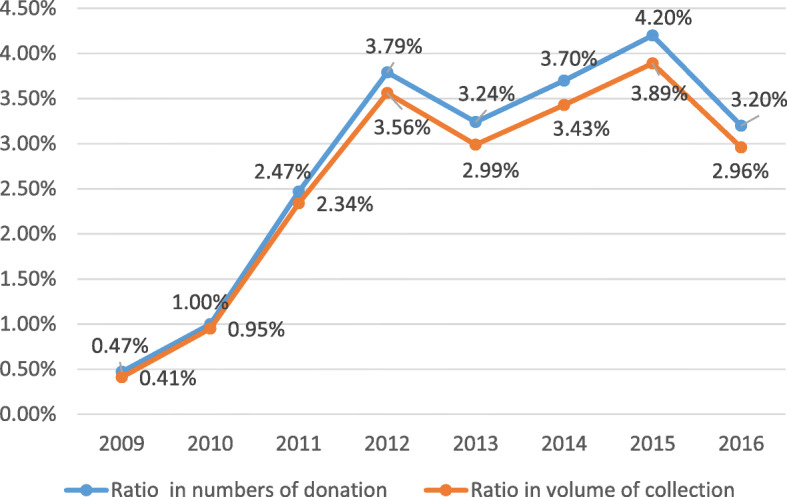
Ratio of mutual blood donation in national blood collection from 2009 to 2016

As a specific mode of donation, it was able to relieve the shortage in clinical blood in China. FRMBD accounted for 0.41% of the national blood collection in 2009 [39]. In 2015, it increased to 4.2% (see Fig. 3) nationwide. In some provinces, it was significantly higher, such as in Hainan (35.6%), Guangxi (25.9%), Xinjiang (11.7%), Gansu (9.5%), and Guangdong (9.3%) [40]. In Xinning City of Guangxi Province, it was up to more than 50% [41], while it was 21% in Beijing in 2017 [42].

However, the rules for mutual blood donation in the Blood Donation Law are too wide in scope for FRMBD and leave room for criminals to sell blood illegally, thereby increasing people’s distrust regarding voluntary blood donation and lowering the quality and safety of donated blood [43]. Private transactions between donors and recipients cannot be supervised by medical institutions or blood centers.

To ensure the quality and safety of blood collection, the NHC issued a government order to cancel FRMBD nationwide, except for some remote areas, by March 2018 [27]. At present, most areas have completely stopped FRMBD. There is no doubt that the cancellation of blood donation imposes a burden on the clinical blood shortage in hospitals [44]. In response to this, VNRBD should be promoted and inter-provincial transfers of blood should be properly allocated to maintain the balance of blood supply and demand.

### Development of blood safety in China

Blood safety, to a large extent, depends on whether the blood source is safe. Ensuring the safety of blood collected is the first step in the process. It took the Chinese government four years to establish and strictly implement the testing procedures, and they have been continuously improving it over the past two decades.

China’s commercial plasma selling emerged in the early 1980s [45]. Since 1993, blood donors have been required to be tested for HIV, hepatitis B, hepatitis C, and syphilis in order to reduce infections through transfusion according to the Health Examination Standards for Blood Donors [32]. However, these regulations were not fully implemented, and HIV-positive individuals could enter the blood plasma collection process without HIV testing. From the end of 1994 to the beginning of 1995, local outbreaks of the infection started occurring in certain provinces including Hebei, Anhui, and Henan. This HIV epidemic was subsequently found to have originated among plasma donors [45]. It was reported that 326 patients from whom blood was donated at plasmapheresis centers in Hebei Province were identified as HIV-positive during 1995–2013. These HIV infections were proven to have started in October 1994 [2].

Finally, the Technical Operation Procedures in Blood Stations standardized donor screening, which has become an essential testing step at blood collection centers since 1997 [46]. From then on, each unit of blood has had to be tested for blood grouping, hemoglobin, alanine aminotransferase (ALT), and HBV surface antigen (HBsAg) before collection. Donated blood (post-collection) has to undergo comprehensive donor testing twice, using different equipment and/or different personnel, including HIV, HBV, hepatitis C virus, ALT, and syphilis [47].

**Fig. 4:**
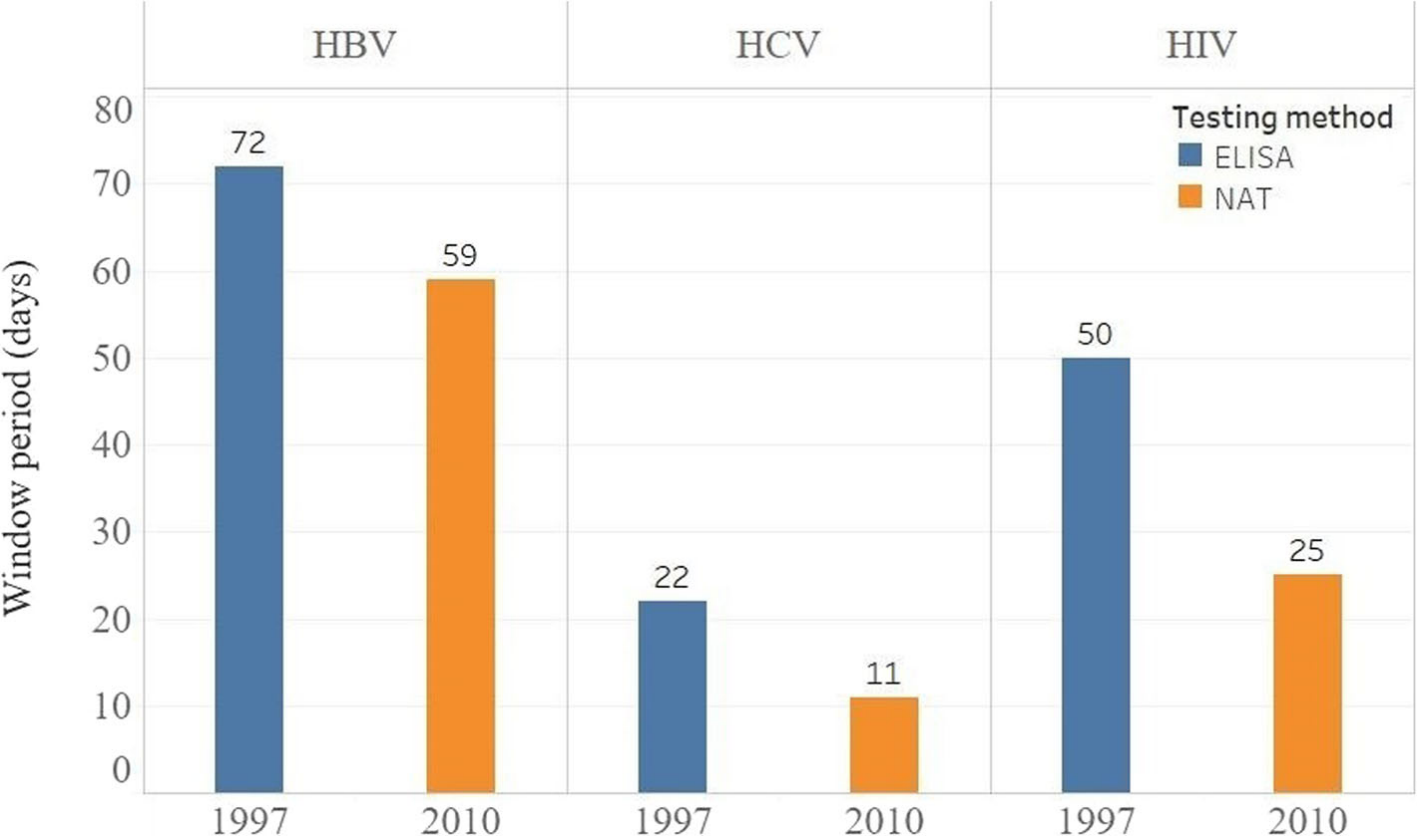
The comparison of window period of HIV, HBV, HCV tested by ELISA & NAT from 1997 to 2010

In the following two decades, testing was strictly implemented. Equipment and technology were updated continuously. For blood group, the RhD type has been mandatorily appraised since 2012 [48]. For serum markers, the colloidal gold strip method was used to detect HBV markers in the early 1990s. Since 1997, serum markers have been tested using an enzyme-linked immunosorbent assay (ELISA) reagent [46]. Since 2010, the Chinese government has established the NAT system in several regions, such as Beijing and Shanghai, covering all types of donations and making great progress in improving blood safety [49]. Due to huge operating costs and the shortage of qualified staff in blood centers, NATs were mainly implemented at the provincial level of blood centers in 2013 [15]. In 2014, the blood tests completed using NAT nationwide approached 4.7 million units, which accounted for 36% of annual blood donations [16]. About 129 million dollars were invested in the nationwide expansion of NAT in 2015 [50]. Eventually, NAT and chemiluminescent immunoassay (CLIA) were formally added to this procedure, according to the Technical Operation Procedures for Blood Centers (2015 Edition) [51]. In order to simplify the procedure and improve its efficiency, serum markers only need to be detected once by ELISA or CLIA except NAT since 2019 [52]. As shown in Fig. 4, the window of HIV, HBV, HCV is shortened when using NAT from 50, 72, and 22 days to 25, 59, and 11 days, respectively [53].

**Fig. 5:**
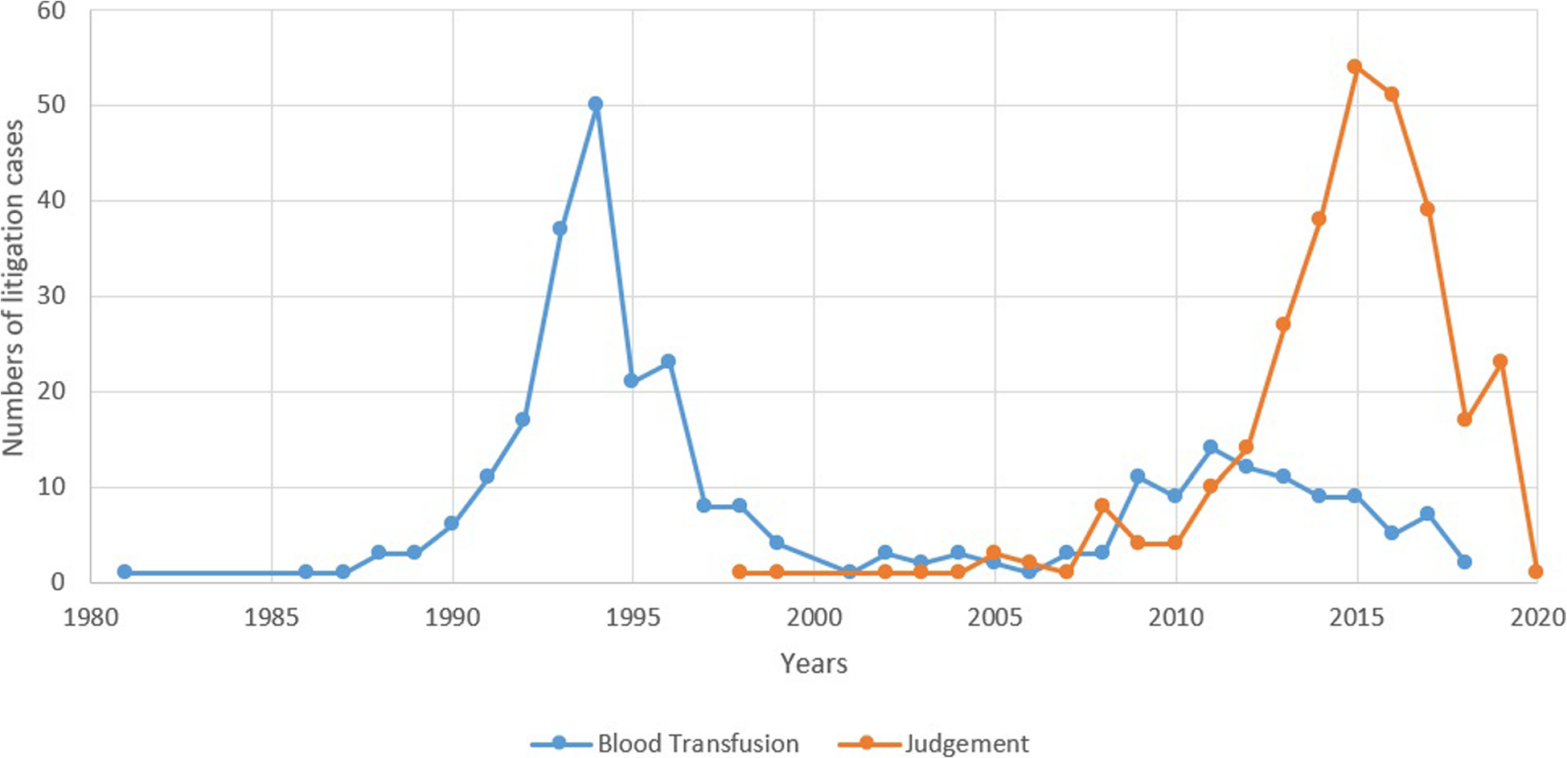
Numbers of litigation cases from 1981 to 2020

By analyzing the litigation related to transfusion from 1981 to 2020, we found that the number of cases experienced a huge reduction, since HCV was required to be tested for in 1994 (Fig. 5). Adverse reactions to blood transfusions accounted for 5.6% of all documents, HBV infections accounted for 5.3%, HIV infections accounted for 11.0%, HCV infections accounted for 76.7%, and syphilis infections accounted for 2.3%. HCV infections took the largest share, 55.8% of which occurred before 1994.

### Transformation of liability in blood transfusion malpractice

Process optimization and technology updates have improved blood safety directly, but the risk of blood transfusion cannot be eliminated completely for some unpredictable factors such as infections, venous thromboembolism [54], transfusion-related lung injury, and transfusion-associated graft-versus-host disease [55]. Therefore, who undertakes this liability is crucial.

Before 1993, there were no regulations related to adverse events caused by transfusion. For instance, medical institutions or blood stations would not be held accountable for HIV infections caused by blood transfusion because the HIV antibody was not required to be tested for until 1993 [46]. Between 1993 and 2002, there were no laws or regulations to compensate for the adverse events caused by blood transfusion, leaving the issue to be addressed by civil law. The judgment results often depended on the opinions of judges and juries.

Between 2002 and 2010, according to Article 33 of the Regulation on the Handling of Medical Accidents, the fault liability principle was deemed applicable in transfusion. It was found that hospitals were not responsible for no-fault transfusion [56], meaning that the hospital does not bear legal responsibility as long as the process meets inspection standards and the technical index, despite the unfavorable consequences caused by infections resulting from blood quality. Moreover, in case of emergency, infections caused by blood transfusion were exempted on the principle that life extension was more important than long-term quality of life. Obviously, this was not fair for the patient who received HIV or HBV infections through transfusion. Thus, in judicial practice, hospitals were ordered to pay compensation patients in cases of infection regardless of whether the transfusion process was in error, for the sake of fairness [57]. Of the 301 cases, 236 hospitals or blood stations in China paid compensation for infections related to transfusion from 1981 to 2018.

After 2010, according to Article 59 of the *Tort Law* of China, damage from transfusion was classified as a special no-fault liability tort. It was the first time it was clearly defined that patients could claim compensation. This principle instituted the protection of the rights and interests of patients. Although the hospital may not be at fault in the whole process, they should assume tort liability for infringing upon a civil right or interest of the patient and pay compensation. As shown in Fig. 5, the number of decisions reached its peak in 2015, which is significantly due to the *Tort Law* of China. Generally, the trial process takes three to 4 years after prosecution. Basically, the patient has the right to claim compensation from the blood center or the hospital if he or she suffered any adverse event due to transfusion or infected blood.

## Discussion

### The legal framework promoting the development of VNRBD

The management of blood donation is an essential part of the public health system and is mainly managed and supervised by the government. Laws and regulations play an important role in blood accessibility and safety. First, the legal framework of blood donation, especially the encouragement of VNRBD, assures the supply for clinical blood use. Second, the legal framework ensures blood safety for all [58]. The rules on blood collection, blood centers, blood tests, and standard operating procedures assure the health rights of blood donors and blood users. Third, a well-established legal framework promotes the uniformity of standards and consistency in the quality and safety of blood and blood products [59].

### Contribution of hospitals in improving blood safety

Blood transfusion therapy is of great significance to the treatment of trauma, anemia, and blood system diseases. Every recipient’s blood group must be determined before transfusion. Application of ABO blood group positive and negative typing and cross-matching tests help avoid acute hemolytic reactions to a large extent. Hemolysis by incompatibility, due to anti-erythrocyte antibodies, remains the most frequent and serious immunological risk to the receiver, especially in a transfusional or feto-maternal context [60]. For some special patients, the Coombs test and an antibody screening test for the detection of irregular antibodies are necessary according to the specifications for clinical transfusion technology [61].

### No-fault liability trend in the protection of patients’ rights in blood transfusion malpractice

The principle of no-fault liability actually protects the interests of patients, given that they are a vulnerable group to a large extent; however, it places a huge economic burden on public service agencies such as hospitals and blood centers. The brief infectious period, before the virus is detected, cannot be completely avoided. Moreover, infection caused by transfusion is generally not detected immediately after transfusion. As a result, loss of blood samples or sample collection errors are commonly found during the investigation of infection cases [62, 63]. In the absence of conclusive evidence, judges would deduce that the blood or blood products offered by hospitals or blood centers were not qualified and standardized if the patient’s family members did not have HCV. Although the retention of blood specimens has been lengthened to 2 years after transfusion, in accordance with the Measures for the Administration of Blood Centers in 2017, it is still difficult to ensure the integrity of the evidence. The final decision depends, largely, on the judge’s inference. In brief, the no-fault liability is a strict liability for hospitals, even though a hospital has the right of recourse against the blood center if it fully complies with transfusion standards.

### Encouraging blood donation and saving clinical blood use to solve the blood supply shortage

It is evident that China has made significant advancements in increasing blood donation. To achieve a self-sufficient blood supply, the WHO states that a minimum of 20 to 25 donors for a population of 1000 is essential [59]. With 15 million donations and a population of 1.39 billion, China has a rate of 11 donors per 1000, far below WHO recommendations and those of high-income countries (39.2 donors per 1000) [40, 64]. The NHC authority expects to reach 15 donors per 1000 by 2020 [65]. There are two main measures to sustain the supply of blood. First, incentive policies are introduced to encourage blood donors. Liu’s research suggests that, aside from promoting public awareness about blood donation, blood traceability during collection, transportation, and storage can increase trust in the relationship between donor and blood center [66]. On the other hand, saving clinical blood is also important to maintain the blood supply; for instance, reducing the number of unnecessary transfusions and increasing pre-operative autologous blood donation.

### Suggestions on the abolishment of mutual blood donation in the blood donation law

In order to prevent blood dealers from profiting from mutual blood donation, the NHC adopted the most direct and effective strategy. Obviously, there are contradictions and conflicts between the requirements and provisions of the NHC in the cancellation of mutual blood donation. First, the cancellation of mutual blood donation has increased the burden of clinical blood shortage while reducing potential transactional crimes between donors, recipients, and blood dealers. Second, mutual blood donation is allowed and institutionalized by the Blood Donation Law; however, the NHC tried to override it by the notice issued in 2018 [27]. The notice is merely a departmental regulation formulated within the authority of the NHC, with a lower rank and limited authority. The inconsistency of laws and regulations makes it difficult to judge the legality of mutual blood donation. Therefore, under the existing legal framework, there are still legal obstacles in terms of the cancellation of FRMBD [67]. The Chinese government has noticed this situation and is ready to take measures. In March 2019, the NHC decided to revise the provisions on the clinical use of blood in medical institutions to cancel mutual blood donation. We believe that the Blood Donation Law will also be revised to cancel mutual blood donation in the future.

## Limitations

Our study on the development of the Legal Framework on blood donation and blood safety in China is based on data collected from Chinese government websites and the WHO database. The amount of blood donation in the same year may vary according to different data sources. Regrettably, data on the volume of donations from 2002 to 2008 were not obtained because they were not publicly available. Furthermore, the judgment documents related to transfusion were collected from the website China Judgments Online. Due to the irregularity of court records in various regions, there may be some omissions in our data.

## Conclusions

The Chinese government institutionalized the voluntary non-remunerated donation principle, enacted regulations for the management of blood transfusion, and adopted advanced blood testing technology to sustain blood supply and ensure blood safety. The number of litigation cases related to transfusion has experienced a huge reduction since the legal framework and testing technology have improved. Despite increased blood donation, however, blood shortages persist. The quality and safety of blood collection can be further improved through the cancellation of mutual blood donation and incentive measures for the voluntary non-remunerated donation of blood, which need facilitation by governmental legislation.

## Data Availability

The datasets used and/or analyzed during the current study are available from the corresponding author on reasonable request.

## Abbreviations

NHC: National Health Commission
NAT: Nucleic acid amplification testing
HIV/AIDS: Human immunodeficiency virus/ acquired immunodeficiency syndrome
HCV: Hepatitis C virus
HBV: Hepatitis B virus
WHO: World Health Organization
VNRBD: Voluntary non-remunerated donation of blood
FRMBD: Family replacement/mutual blood donation
